# Cross-Cultural Adaptation and Validation of the Malayalam Version of the Vocal Tract Discomfort Scale

**DOI:** 10.3390/diagnostics15030259

**Published:** 2025-01-23

**Authors:** Sunil Kumar Ravi, Srushti Shabnam, Saraswathi Thupakula, Vijaya Kumar Narne, Krishna Yerraguntla, Abdulaziz Almudhi, Irfana Madathodiyil, Feby Sajan, Kochette Ria Jacob

**Affiliations:** 1 Department of Medical Rehabilitation Sciences, College of Applied Medical Sciences, King Khalid University, Abha 61481, Saudi Arabia; 2 Speech-Language Pathology Unit, College of Applied Medical Sciences, King Khalid University, Abha 61481, Saudi Arabia; 3 Nitte Institute of Speech and Hearing, NITTE University, Mangaluru 575018, India; 4 Shravana Institute of Speech and Hearing, Ballari 583104, India

**Keywords:** Vocal Tract Discomfort Scale (VTDS), Voice Handicap Index (VHI), normophonic, dysphonic, voice disorders, Malayalam

## Abstract

**Background:** Voice disorders significantly impact individuals’ physical, functional, and emotional well-being, necessitating comprehensive assessment tools. The Vocal Tract Discomfort Scale (VTDS) assesses the frequency and severity of vocal discomfort symptoms. Despite its global adaptations, no validated Malayalam version has existed. This study aimed to adapt and validate the VTDS for Malayalam speakers (VTDS-M). **Method:** The study was conducted in two phases: Phase I involved translation and cultural adaptation of VTDS into Malayalam, followed by content validation by native-speaking speech language pathologists; Phase II involved validation of VTDS-M on 150 professional voice users, categorized into normophonic (*n* = 105) and dysphonic (*n* = 45) groups based on otolaryngological and perceptual voice evaluations. Participants completed VTDS-M and VHI-M (Voice Handicap Index—Malayalam). **Results:** The results showed strong internal consistency (Cronbach’s α = 0.827 for frequency, 0.813 for severity). Significant differences were observed between groups for VTDS-M subscales and total scores, confirming its discriminatory capability. ROC analysis established a cut-off score of 11.5, with an AROC of 0.749, 64.4% sensitivity, and 79.0% specificity. Also, VTDS-M correlated positively with VHI-M, especially the physical and emotional subscales. **Conclusions:** VTDS-M demonstrated reliable psychometric properties and diagnostic accuracy, making it a valuable tool for assessing vocal discomfort in Malayalam-speaking populations specifically among the professional voice users. Future studies should explore its applicability to non-professional voice users with varied severity levels of dysphonia.

## 1. Introduction

The voice is a multifaceted phenomenon needed for verbal communication and the expression of emotional and creative emotions. Therefore, voice disorders can have a detrimental impact on the physical, functional, and emotional components of a patient’s quality of life. Voice problems arise from a variety of causes, including hyperfunctional, organic, and psychogenic disorders, which can influence the acoustic and perceptual attributes of an individual’s voice. A thorough assessment of a patient with a voice issue encompasses perceptual analysis, acoustic analysis, laryngeal imaging, and the incorporation of patient-reported outcome measures (PROMs). Ongoing research aims to develop novel acoustic measurements, acoustic indices, perceptual analysis rating scales, and imaging technologies to enhance patient care. The PROMs differ from the perceptual analysis measures evaluated by clinicians, as PROMs represent the patient’s perspective of their voice difficulty.

This emphasizes the significance of including psychosocial assessment as a crucial component of voice assessment [1,2]. Typically, these psychosocial assessment tools/voice-related quality of life measurements gauge the patient’s evaluation of their voice problems. The Vocal Tract Discomfort Scale (VTDS) [3], Voice Handicap Index (VHI) [4], Voice-Related Quality of Life (V-RQOL) [5], Voice Activity and Participation Profile (VAPP) [6], and Voice Symptom Scale (VoiSS) [7] have been frequently used to assess the impact of voice disorders on a patient’s quality of life, providing valuable insights into the patient’s perception of the disorder. These methods provide the quantification of the psychosocial consequences of voice dysfunction and evaluate the efficacy of the intervention. The VTDS [3] was developed to evaluate the frequency and severity of discomfort experienced by patients concerning eight specific symptoms. Initially, it was validated on patients with muscle tension dysphonia and subsequently adapted and validated in various languages, including Persian [8], Italian [9], Arabic [10], Argentine [11], German [12], Korean [13], and Brazilian [14].

Vocal discomfort in various populations has been evaluated using VTDS and the studies by Rodrigues et al. [15], Amaral et al. [16], Limoeiro et al. [17], Galletti et al. [18], and Porto et al. [19] have demonstrated the effectiveness of VTDS as a tool for identifying symptoms of vocal tract discomfort in teachers. In their study, Galletti et al. [18] found a moderate correlation between VTDS and VoiSS. They also demonstrated that VTDS was effective in identifying symptoms of vocal tract discomfort in teachers who were at a higher risk. Amaral et al. [16] discovered that teachers experienced more discomfort in their vocal tract after the day of teaching. Research has demonstrated that VTDS is an efficient tool for identifying symptoms of vocal tract discomfort in other professional voice users, such as singers and dramatic actors [20,21,22].

Furthermore, Tohidast et al. [23] conducted a study to examine the vocal quality and vocal tract discomfort symptoms in individuals with COVID-19 using GRBAS and VTDS. The findings indicated that COVID-19 patients exhibit greater abnormalities in voice quality compared to healthy individuals. In addition, individuals with COVID-19 tend to have more frequent and severe physical discomfort in their vocal tract compared to healthy participants. It is also usual for these patients to report minor vocal tract discomfort. VTDS was found to be effective in identifying the severity and frequency of vocal tract discomfort in individuals who are undergoing thyroidectomy [24]. The published literature to date indicates that VTDS is a dependable instrument for detecting symptoms of vocal tract discomfort across various professional users and diverse voice disorders.

India is known for its cultural and linguistic diversity, with 22 official languages and a large number of dialects spoken across 140 million people. Malayalam is a Dravidian language that is spoken by more than 35 million individuals in the Indian state of Kerala. Prior research conducted by Devadas et al. [25] and Menon et al. [26] revealed a greater incidence of voice abnormalities among teachers (45%) and priests (25.2%). However, there are just two self-evaluation measures for assessing voice quality that have been culturally adapted and validated in the Malayalam language. These scales are the Voice Handicap Index (VHI-M) [27] and the Vocal Fatigue Index (VFI-M) [28]. The Vocal Handicap Index (VHI) primarily evaluates the functional, physical, and emotional dimensions of the patient, whereas the Vocal Fatigue Index (VFI) measures the levels of vocal exhaustion specifically in professional voice users.

Voice disorders can result from multiple causes such as organic, functional, hyperfunctional, and many others, with each disorder presenting with different symptoms such as changes in the structure and function of vocal folds or symptoms limited only to the changes in voice production without structural changes to the vocal folds. Although these symptoms are the most common complaints to speech language pathologists or otolaryngologists, the individual with the voice disorder often experiences a wide range of vocal discomfort such as pain, dryness, feeling of a lump in the throat, sore throat etc., which needs to be addressed by the treating physician. These symptoms of discomfort are an important treatment outcome measure from the perspective of the individual with the voice disorder to assess the efficacy of treatment. The VTDS has been widely used to assess these signs and symptoms of vocal discomfort among individuals with various voice disorders across the world using the adapted versions of VTDS. The VTDS has many advantages, such as it is simple with only eight symptoms which are most commonly reported by individuals with voice disorders, and is measured on both frequency and severity on a 7-point Likert scale which makes it easy for any lay person to rate their level of vocal tract discomfort. Another advantage of the scale includes the short time taken to complete the rating of the scale compared to other scales such as VoiSS as reported by Lopes et al. [29].

The review of the literature clearly implies the lack of established research in Indian languages, including in Malayalam, although there have been studies showing the increased prevalence of voice disorders by Devadas et al. [25] and Menon et al. [26] among Malayalam-speaking professional voice users. Currently, only VHI-M [27] and VFI-M [28] are the available patient reported outcome measures available in the Malayalam language. The VTDS, which has several advantages with high accuracy of reporting patient perceived discomfort in vocal tract subjectively has not been adapted and validated for use in the Malayalam language, which may affect the overall comprehensive clinical evaluation of individuals with voice disorders. Therefore, the objective of this work is to adapt and validate the VTDS specifically for the Malayalam language speaking professional voice users.

## 2. Method

The present study was carried out using a cross-sectional method in two phases with Phase I consisting of the adaptation of VTDS in Malayalam, and Phase II consisting of validation of VTDS-M on normophonic and dysphonic groups. The study was approved by the Institute Research Ethics Committee with approval no. ECM#2023-1805.

### 2.1. Phase I

The VTDS was translated into the Malayalam language by two native speakers of Malayalam and a linguist. The translated scale was given to three independent Malayalam-speaking speech language pathologists who are native Malayalam speakers and who were not part of the study, and they were asked to rate the translation in terms of appropriateness of language and word structure and also to do a back translation to the English language. Based on the feedback provided by the native speech language pathologists, a final VTDS was prepared in the Malayalam language (Appendix A). During the translation phase, some obstacles arose, particularly in identifying equivalent terms in Malayalam for the English VTDS. In the Malayalam language, there exists a subtle distinction between “aching” and “sore”. The challenges were resolved following discussions with native Malayalam linguists, leading to the finalization of the translation.

### 2.2. Phase II

A total of 150 professional voice users (teachers, priests) in the age range of 26 to 58 years with Malayalam as a mother tongue were randomly selected from Kottayam district of Kerala, a southern state in India. A questionnaire was prepared and administered to all the participants to collect the demographic data, voice usage, history of voice disorders, surgeries undergone, etc., following which all the participants were assessed using GRBAS and Voice Handicap Index—Malayalam by the speech language pathologist. A total of 45 participants were found to be having organic and/or hyperfunctional voice disorders which were later confirmed by the otolaryngologist. Based on the otolaryngological examination and results of perceptual evaluation of voice, 105 participants were categorized under the normophonic group and 45 participants were categorized under the dysphonic group. The power analysis was carried out using G-power software 3.1.9.7 and the normophonic and clinical group required 42 subjects in each group at an alpha level of 0.05 in the *t*-test, 0.65 in effect size, and 0.9 in power. The participants of the study were included in the study with the exclusion criteria being the presence of any disorders of neurological origin, endocrinal, or psychological. The adapted VTDS-M was given to the participants of both groups to rate the self-perceived vocal discomfort. For VTDS-M, participants were instructed to rate their discomfort on a 7-point Likert scale from ‘0’ to ‘6’, where ‘0’ indicates absence of symptom and ‘6’ indicates always/extreme about the frequency and severity of their discomfort on eight symptoms. Accordingly, the scores will range from 0 to 48 for each of the frequency and severity domains. The lower scores on VTDS indicate either normal or mild vocal discomfort, and higher scores indicate severe vocal discomfort.

### 2.3. Statistical Analysis

The Shapiro–Wilk test was employed to evaluate data normality. Descriptive statistics were utilized for each domain of the VTDS, the overall VTDS score, as well as for the VHI (functional, physical, and emotional subscales) and the total VHI score. Cronbach’s α analysis assessed the reliability of subsections of frequency and severity of VTDS—Malayalam, whereas an independent sample *t*-test compared the results of normophonic and dysphonic groups for both VTDS and VHI scales. An ROC analysis was conducted to assess the diagnostic accuracy (sensitivity and specificity) of VTDS—Malayalam using a designated cut-off score. A Spearman’s correlation analysis was conducted to assess the relationship between the domains and total scores of VTDS—Malayalam and VHI—Malayalam. The statistical analysis was conducted using IBM SPSS Statistics version 22.0 software.

## 3. Results

### 3.1. Internal Consistency of VTDS-M

The Cronbach’s α values for the VTDS-M showed good reliability for the subscale of frequency and severity, and totaled at 0.827, 0.813, and 0.905 respectively.

### 3.2. VTDS-M and VHI-M Scale Scores

**Figure 1:**
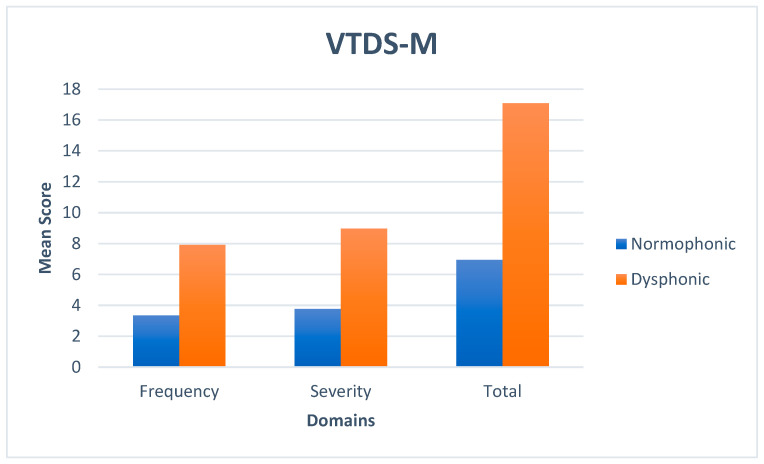
Mean scores of VTDS-M for normophonic and dysphonic groups.

The data were analyzed using SPSS v22.0 software for calculating the mean and standard deviations of scores (Table 1) of subsections and total scores of VTDS-M and VHI-M, and the scores were compared between normophonic and dysphonic groups using the independent sample *t*-test. The descriptive statistics revealed that the dysphonic group exhibited higher scores across both domains of the VTDS-M and also in the overall total indicating greater voice-related discomfort compared to the normophonic group. Similarly on VHI-M, the dysphonic group revealed a significant handicap due to voice problems compared to the normophonic group. The results of the statistical analysis showed significant differences between the normophonic group and dysphonic group for the frequency subscale [t(148) = 5.765, *p* < 0.05]; severity subscale [t(148) = 5.096, *p* < 0.05]; and for the VTDS-M total scores [t(148) = 5.696, *p* < 0.05]. The results of statistical analysis revealed a significant difference in the functional domain [t(148) = 3.862, *p* < 0.05]; physical domain [t(148) = 5.961, *p* < 0.05]; emotional domain [t(148) = 4.255, *p* < 0.05]; and for total scores of VHI-M [t(148) = 6.258, *p* < 0.05]. Therefore, for both the scales, the dysphonic group had significantly higher scores compare to the normophonic group, indicating voice impairment and vocal tract discomfort. The graphical representation of mean scores are depicted in Figure 1.

### 3.3. Diagnostic Accuracy of VTDS-M

**Figure 2:**
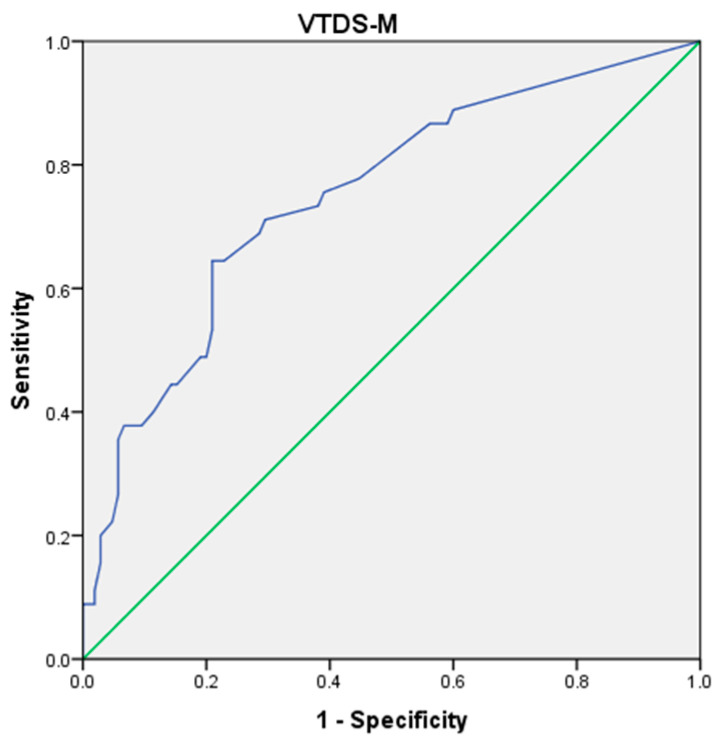
Results of ROC analysis for VTDS-M.

The ROC analysis for VTDS-M indicated that the VTDS-M value of 11.5 and less can be considered as no vocal discomfort, while values above 11.5 can be considered as the presence of vocal discomfort (Table 2). The A_ROC_ values of 0.749 with a *p* value less than 0.001 (Figure 2) offer good accuracy in discriminating individuals with and without vocal discomfort. Further, 64.4% sensitivity, 79.0%, and Youden index of 0.434 indicates that VTDS-M possesses good precision in discriminating individuals with and without vocal discomfort.

### 3.4. Relationship Between VHI-M and VTDS-M

The correlations between the VTDS-M and the VHI-M are shown in Table 3. The scores for the subscales of frequency, severity, and total scores of VTDS-M showed a positive moderate correlation with the physical subscale, low positive correlation with the emotional subscale and total score; and negligible correlation with the functional subscale of the VHI-M.

## 4. Discussion

The current study translated the VTDS into the Malayalam language (VTDS-M) and the relationship between VTDS-M and VHI-M. It was further used to examine characteristics of vocal tract discomfort in individuals with and without voice disorders.

### 4.1. Cross-Cultural Translation and Adaptation

The significance of self-rating scales and quality of life scales is rising in diagnostic assessment and measurement of treatment outcomes for people with voice problems worldwide. Nonetheless, there exists a scarcity of self-rating scales and quality of life assessments for patients with voice disorders in the Indian context, particularly in the Malayalam language, which is spoken by over 35 million individuals both within and outside the state of Kerala. As of now, only two scales have been adapted into Malayalam: the Voice Handicap Index—Malayalam [27] and the Vocal Fatigue Index [28]. While both the VHI and VFI aim to assess the impact of voice problems on quality of life and self-perceived vocal fatigue, each scale has specific limitations. These scales do not provide a detailed assessment of the specific sort of discomfort reported by individuals with vocal disorders. Consequently, the VTDS has been recognized as a suitable instrument for assessing discomfort levels and has been translated and adapted into various languages [8,9,10,11,12,13,14], and the modified versions demonstrated strong internal consistency and validity in assessing the frequency and severity of vocal discomfort in individuals with voice problems.

Phase 1 of the study was conducted in accordance with the guidelines established by Beaton et al. [30], for the cross-cultural translation and adaption of the VTDS into the Malayalam language, in order to minimize bias. The linguistic and cultural factors were minimally addressed during the translation of the scale into Malayalam, which was subsequently evaluated through a content validation process conducted by native Malayalam-speaking speech language pathologists.

### 4.2. Internal Consistency of VTDS-M

The Cronbach’s α values of 0.827 and 0.813 for the frequency and severity subscales of the VTDS-M demonstrate good internal reliability. This indicates that the items within each subscale have a high degree of correlation, suggesting they consistently measure the same underlying construct. Similar findings have been previously reported in prior studies across several languages, including Persian [8], Italian [9], Arabic [10], Argentine [11], German [12], and Korean [13].

### 4.3. Diagnostic Accuracy of VTDS-M

The ROC analysis was carried out to measure the optimal threshold for VTDS-M using the Youden index, which ranges from 0 to 1 in which 1 signifies perfect diagnostic accuracy, while a score of 0 indicates the test’s ineffectiveness. The Youden index is calculated by evaluating the sensitivity and specificity across all potential cut-off points [31]. The results underscore the efficacy of the VTDS-M as a dependable and precise instrument for evaluating vocal discomfort. The established cut-off value of 11.5 serves as an accurate criterion for differentiating persons with vocal discomfort from those without, with scores beyond this threshold signifying the existence of vocal discomfort. The A_ROC_ value of 0.749, with a significant *p*-value below 0.001, sensitivity of 71.1%, and specificity of 70.5%, indicates strong overall accuracy in distinguishing between those with and without voice pain. However, the determined cut-off value of 11.5 is significantly lower than the cut-off scores reported by other studies, specifically 26 for VTDS—Arabic [10] and 25.75 for VTDS—German [12]. This may be attributed to the selection of a population in the current study that exclusively comprised patients with milder dysphonia. The diagnostic accuracy (sensitivity and specificity) of VTDS-M may be inferior to other studies, potentially due to the milder dysphonic group and the subtle distinctions between certain items, which made it challenging for participants to differentiate, such as sore versus aching, tickling, etc. Future research is necessary on a large population with moderate and severe dysphonia to assess the diagnosis accuracy for severe cases.

### 4.4. Performance of Dysphonic and Normophonic Groups on VTDS-M and VHI-M Scale Scores

The statistical analysis comparing the scores of the VTDS-M and VHI-M between normophonic and dysphonic groups revealed significant variations in perceptions and sensations of vocal discomfort and voice impairment. The VTDS-M results indicate substantial disparities between the normophonic and dysphonic groups across all assessed parameters. The dysphonic group demonstrated markedly elevated scores on the frequency subscale, severity subscale, and overall scores on the VTDS-M in comparison to the normophonic group. The data indicate that patients with voice impairment have a greater frequency and intensity of vocal discomfort. In the VTDS-M, the dysphonic group reported elevated scores for frequency and severity of dryness, followed by soreness and irritability. This finding contradicts the study by Santi et al. [11], which identified the sensation of a lump in the throat as the most frequently reported discomfort in the clinical group, potentially attributable to variations in vocal pathologies. While the original VTDS was validated for individuals with muscle tension dysphonia, the findings of the current study suggest that the adapted VTDS-M can be utilized clinically to assess vocal discomfort in patients with voice disorders and teachers, as reported by Galletti et al. [18].

The VHI-M results reveal substantial disparities between the two groups in all domains (functional, physical, and emotional) and in the overall scores. This indicates that persons in the dysphonic group experience more significant functional limitations, physical discomfort, and emotional distress associated with their voice than those in the normophonic group. The mean ± SD values of VTDS-M in this study aligned with those from prior research on the normophonic group [8,9,10,13]. The mean ± SD values of VTDS-M for the dysphonic group were comparatively lower than those reported in previous studies [8,9,13], with the exception of the Arabic version of VTDS [10]. This can be ascribed to the reduced severity of vocal issues in the dysphonic cohort, as the majority of participants in this group exhibited a lesser degree of dysphonia in the current study.

### 4.5. Relationship Between VHI-M and VTDS-M

Firstly, the positive moderate correlation between the VTDS subscales of frequency, severity, and total scores with the physical subscale of the VHI indicates that individuals who report higher levels of vocal tract discomfort tend to also experience more physical limitations related to their voice. This suggests that discomfort stemming from vocal tract issues, such as soreness or tension, may directly impact physical aspects of voice production. Additionally, the low positive correlation observed between the VTDS subscales and the emotional subscale and total score of the VHI suggests that vocal tract discomfort may contribute to emotional distress related to voice function, albeit to a lesser extent. On the other hand, the negligible correlation between the VTDS subscales and the functional subscale of the VHI indicates that vocal tract discomfort may not significantly impact perceived functional limitations in voice use. The items in the VTDS-M primarily address the physical discomfort experienced by individuals with voice disorders, and the physical discomfort may not be directly correlated with functional handicap as the physical discomfort may not always affect the functional abilities in daily activities of living of an individual. This suggests that while individuals may experience physical discomfort or emotional distress related to vocal tract issues, they may not necessarily perceive limitations in their ability to perform functional tasks requiring voice use. Further, the low correlation can also be due to the reason that the participants in the dysphonic group have milder forms of dysphonia which may not have a significant effect on voice handicap. We hypothesize that there is a positive strong correlation between VTDS and VHI when these scales are administered to moderate and severe dysphonia patients. However, the results of the present study contradict the results of a study carried out on 333 Flemish participants by Luyten et al. [32], who reported a low correlation between frequency and severity of VTDS and VHI total scores. This is probably due to differences in the population on which the study was carried out where the study by Luyten et al. [31] was carried out on the Flemish population without self-perceived voice disorders, whereas the present study was carried out on individuals with hyperfunctional voice disorders. Further, Lopes et al. [29] have reported a moderately positive correlation between VTDS and VoiSS scales among individuals with muscle tension dysphonia and also reported that pharyngeal symptoms are strong indicators for voice disorders [29]. Additional studies are required to evaluate the correlations between VTDS and other self-perceived rating scales in future to establish the validity of the VTDS to be able to use it in clinical assessment of voice disorders.

## 5. Conclusions

The VTDS-M allows patients to report vocal discomfort across eight symptoms typically linked to voice disorders, and can be employed in standard clinical evaluations of such diseases. The Malayalam adaptation of the Vocal Tract Discomfort Scale demonstrated strong internal consistency and exhibited commendable diagnostic accuracy with a cut-off score of 11.5 among professional voice users. Consequently, the VTDS-M serves as a dependable supplementary self-rating scale in the thorough diagnostic evaluation of voice problems. The VTDS-M serves as a dependable instrument for evaluating therapy outcomes in patients with voice disorders, as patients readily recognize feelings of vocal discomfort. The current study focused on professional voice users with mild dysphonia resulting in limited generalizability of results; hence, future research could include non-professional voice users with moderate and severe dysphonia with different voice pathologies.

## Figures and Tables

**Table 1:** Mean and SD Scores of VTDS-M and VHI-M for normophonic and dysphonic groups.

Scale	Domains	Normophonic Group		Dysphonic Group	
		Mean	SD	Mean	SD
VTDS-M	Frequency	3.352	3.95	7.911	5.41
	Severity	3.771	5.02	8.977	7.13
	Total	6.952	8.56	17.089	12.73
VHI-M	Functional	3.281	2.80	5.533	4.33
	Physical	2.15	3.46	7.133	6.75
	Emotional	0.342	1.099	2.044	3.75
	Total	5.733	5.79	14.822	12.00

**Table 2:** Sensitivity and specificity of VTDS-M.

Scale	Cut-Off Values	A_ROC_	Sensitivity	Specificity	Youden Index
VTDS-M	11.5	0.749 **	64.4%	79.0%	0.434
VTDS-M Frequency	5.5	0.748 **	62.2%	77.1%	0.393
VTDS-M Severity	5.5	0.743 **	64.4%	78.1%	0.425

**: *p* < 0.01.

**Table 3:** Correlations between the VTDS-M and VHI-M scores.

VTDS-M	VTDS-M			VHI-M			
	Frequency	Severity	Total	Functional	Physical	Emotional	Total
Frequency	1	0.896 *	0.954 *	0.261 *	0.525 *	0.368 *	0.499 *
Severity	0.896 *	1	0.942 *	0.187	0.546 *	0.378 *	0.488 *
Total	0.954 *	0.942 *	1	0.240 *	0.584 *	0.420 *	0.536 *

*: *p* < 0.05.

**Figure A1:**
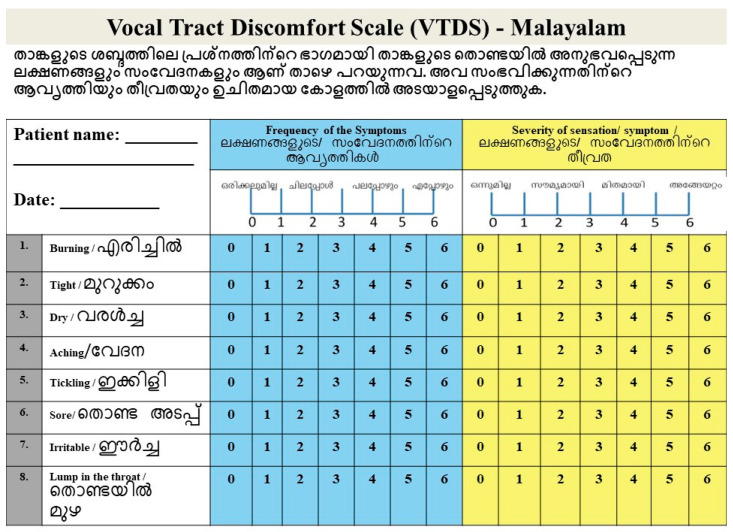
Malayalam version of the VTDS.

## Data Availability

Data are available with the corresponding author mentioned in this research paper.
